# Strand 1A variant in neuroserpin shows increased aggregation and no loss of inhibition: implication in ameliorating polymerization to retain activity

**DOI:** 10.1042/BSR20221825

**Published:** 2022-12-16

**Authors:** Shoyab Ansari, Shahzaib Ahamad, Abdul Burhan Khan, Sana Fatima, Tahif Ahmad, Yasmeen Khan, Dinesh Gupta, Mohamad Aman Jairajpuri

**Affiliations:** 1 Protein Conformation and Enzymology Laboratory, Department of Biosciences, Jamia Millia Islamia (A Central University), New Delhi 110025, India; 2 Translational Bioinformatics Group, International Centre for Genetic Engineering and Biotechnology (ICGEB), New Delhi, 110067, India; 3 CSIR-Institute of Genomics and Integrative Biology, Mathura Road, New Delhi 110025, India

**Keywords:** epilepsy, neuroserpin, serpin, tissue plasminogen activator

## Abstract

Neuroserpin (NS) is predominantly expressed in the brain and is the primary inhibitor of tissue plasminogen activator (tPA). NS variants are associated with the neurogenerative disease termed familial encephalopathy with neuroserpin inclusion bodies (FENIB). The disease is characterized by variable age of onset and severity. The reactive center loop (RCL) insertion-based inhibitory mechanism of NS requires a coordinated conformational change leading to a shift in the strands of the β-sheet A and movement of helix F. Strand 1A is connected to the helix F at its C terminal end and with the strand 2A at its N terminal, both these domain move for accommodating the inserting loop; therefore, a variant that influences their movement may alter the inhibition rates. A molecular dynamic simulation analysis of a H138C NS variant from strand 1A showed a large decrease in conformational fluctuations as compared with wild-type NS. H138 was mutated, expressed, purified and a native-PAGE and transmission electron microscopy (TEM) analysis showed that this variant forms large molecular weight aggregates on a slight increase in temperature. However, a circular dichroism analysis showed its secondary structure to be largely conserved. Surprisingly, its tPA inhibition activity and complex formation remain unhindered even after the site-specific labeling of H138C with Alexa fluor C_5_ maleimide. Further, a helix F-strand 1A (W154C-H138C) double variant still shows appreciable inhibitory activity. Increasingly, it appears that aggregation and not loss of inhibition is the more likely cause of shutter region-based variants phenotypes, indicating that hindering polymer formation using small molecules may retain inhibitory activity in pathological variants of NS.

## Introduction

**Figure 1:**
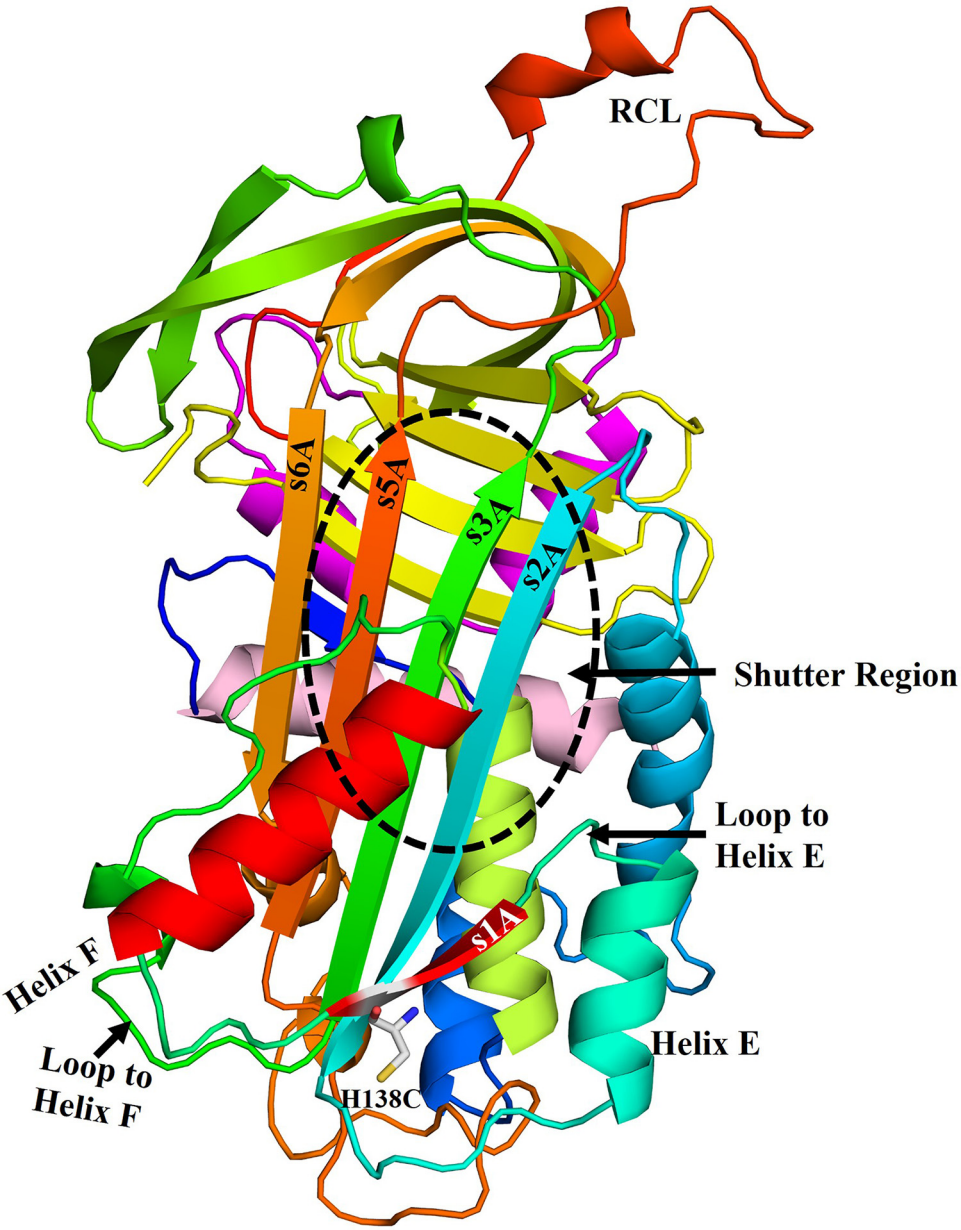
Schematic view of native neuroserpin The structure of the human neuroserpin (3F5N) shows the position of helix F and H138C residue on strand 1 of β-sheet A. The region which undergoes conformational change on the insertion of the reactive center loop as strand 4A is depicted as the shutter region. The picture was created by using PyMOL molecular graphic system.

Neuroserpin is a member of the serpin (serine protease inhibitor) superfamily that is expressed mostly in the developing and adult nervous system, where it primarily acts as a tissue plasminogen activator (tPA) inhibitor [1,2]. The expression of neuroserpin is associated with several physiological processes including synaptogenesis, embryonic development, regeneration, cell morphology and neurogenesis [3–7]. Some variants of neuroserpin trigger aberrant conformation that may lead to a neurological disease termed as FENIB [8]. FENIB symptoms include presenile dementia and cognitive impairment due to neurodegeneration [9,10]. NS consists of an exposed reactive centre loop (RCL) which acts as bait for the tPA and forms a complex, which leads to the cleavage and reactive site loop (RCL) insertion into the β-sheet A [11–13]. During the protease inhibition opening of strands 3A and 5A of central β-sheet A is required for the insertion of RCL as a strand 4A [9]. This region of β-sheet A that allows the insertion of RCL is known as the ‘shutter region’ and is shown in Figure 1, which consists of strands 3A, 5A and helix F [14,15]. Variants in or near the shutter region of NS like S49P, S53R, H338R, G392E, G392R and L47P result in pathological phenotype that are with different severity and the rate of polymerization [16,17]. NS has also been shown to be with unusual chaperone selectivity that is involved in the amyloid inhibition [18]. Polymeric NS causes neuronal degeneration via mitochondrial alterations that also affect the communication between mitochondria and ER [19]. Embelin was shown to bind to the human NS and impairs its polymerization [20]. Retention of inhibitory activity on hindrance in the polymerization mechanism will be extremely effective in reducing the pathological phenotype.

NS intracellular accumulation of inclusion bodies has been directly linked with increased neurotoxicity with pathological phenotype [21]. A similar inclusion type accumulation is also shown to be associated with serpins like α1-antitrypsin, antithrombin, α1-antichymotrypsin and C1 inhibitor [22–25]. During the NS inhibition mechanism, the insertion of RCL into the β-sheet A requires conformational changes within its core structure including strands 1, 2, 3 and 5 of β-sheet A and helix F (Figure 1) [26–30]. A downward displacement of helix F toward strand 3A was observed in the G117F mutant of α1 antitrypsin due to phenylalanine side-chain repacking at the hydrophobic interface of the helix F and β-sheet A [31]. In contrast, restructuring at the upper part of helix F in α1 antitrypsin was found to be necessary during RCL insertion [32]. Similarly, principal component analysis after the NS simulation study reveals the movement of helix F away from the β-sheet A [12,33].

Strand 1A (s1A) interaction in serpins may influence the conformational changes in the helix F and loop connecting helix F to s3A [34–38]. Both helix F and strand 3A are part of shutter region, histidine residue at 138 position is present at the lower pole of strand 1A (Figure 1). C-terminal of s1A connects to helix F through a loop and its N-terminal end connects to the s2A via helix E. Due to its unique position to influence the movement of both helix F and s2A during RCL insertion, s1A role in aggregation behavior and tPA inhibition mechanism needs to be deciphered. We explored the dynamics of conformational changes in s1A by generating its cysteine variant (H138C) and show that tPA-dependent exposure does not affect tPA complex formation or inhibition mechanism, but the aggregation propensity is significantly increased. Therefore, slowing the rate of aggregation with the use of small molecules may retain significant NS inhibitory activity in the variants near or in the shutter region.

## Materials and methods

### Molecular dynamics simulation

Molecular dynamics (MD) simulations studies were initiated by obtaining the molecular coordinates of WT NS from the PDB (3FGQ) file [39], and the missing RCL loop coordinate was modeled by using a Swiss model online server [40]. About 50 ns MD simulations of WT NS, H138C and W154C-H138C were performed using GROMACS 5.1.5 software package installed on high-performance computing (HPC) Dell server with 128GB RAM, and CUDA enabled NVIDIA (Model: Nvidia Tesla V100) graphics processing units (GPUs) (GPU Memory Total 16 GB X 4) [41]. CHARMM force-field was used to create topology parameter files for native protein [42]. To determine the intermolecular (non-bonded) potential, the particle mesh Ewald approach was used which is represented as the sum of the long-range electrostatic force, Lennard–Jones (LJ) force and pairwise Coulomb interaction. Numerical integrations were determined by using the velocity Verlet algorithm. Maxwellian distribution at the given absolute temperature was used to generate the initial atomic velocities followed by the immersed system with SPC/E water model and further center of cubic grid box was used to place protein (1.0 nm) [43]. Conjugate gradient (CG) algorithms were used for the energy minimization by utilizing a convergence criterion of 0.005 kcal mol^−1^ Å^−1^. Separate NVT and NPT ensemble conditions were used for the two-standard equilibration phase. To maintain the constant temperature and the pressure (1 bar and 300 K) of the system Berendsen thermostat and Parinello–Rahman barostat were used. The leap-frog algorithm with a 2 fs time step was applied for integrating the equation of motion with the periodic boundary condition (PBC). The relaxation of the grid box with water along with the counterions was used to achieve for production of the fixing of constraints. Additionally, simulation was performed at 50,000 ps (50 ns) totaling 150 ns for this study.

### Site-directed mutagenesis

A point mutation in WT NS plasmid was introduced by using complementary oligonucleotide primer design by *Primer X* tools (http://www.bioinformatics.org/primerx/). Quick change II mutagenesis kit (Stratagene) was used for the site-directed mutagenesis of H138C and W154C-H138C using PCR amplification. In the first PCR step, 50 µl of reactant mixture was prepared to contain 1X reaction buffer, 200 µM dNTPs, 0.1–0.2 µM of corresponding mutation primer, 2–10 ng of DNA template (WT NS and W154C-H138C) and 1 µl of *PfuUltra* HF DNA polymerase (2.5 U/µl). Mutagenic primer of strand 1A and helix F are listed in Supplementary Figures S2 and S4. PCR based amplification proceeds through a cycle of denaturation at 94°C (40 s), annealing at 55°C (40 s) and extension at 72°C (8 min) for a total of 18 cycles followed by final extension of the last cycle at 72°C (10 min). Both mutations were confirmed by DNA sequencing. PCR product was digested with methylated sensitive endonuclease *Dpn*I enzyme. Undigested non-methylated daughter plasmid DNA was used for transformation in DH5α strains of *Escherichia coli* for plasmid amplification [44].

### Expression and purification

For NS expression and purification, *E. coli* BL21 (DE3) competent cells (Novagen) was transformed with PET28b plasmid carrying the human NS gene with N-terminus 6-His tag. Briefly, bacterial cells were grown in LB Broth (Himedia) containing 100 µg/ml kanamycin and protein expression was induced with 1 mM isopropyl-β-d-thiogalactopyranoside at 17°C for 16–18 h. Cell pellet was lysed with lysis buffer [30 mM Tris-HCl (pH 8.0), 5 mM imidazole, 150 mM NaCl and 0.1% (v/v) Triton X-100, 5% (v/v) glycerol, 0.1 mg/ml lysozyme and 100 µM PMSF] followed by the 30 min incubation in ice. Under continuous cooling, the cell lysate was sonicated for 5 min with 10 s pulses at 40% amplitude. The cell lysate was clarified by centrifugation at 9000 × ***g*** for 10 min and filtered. Soluble fraction was loaded onto a Ni-NTA agarose resin column (GE Healthcare) for purification. The Ni-NTA resin column was washed with 30 ml of salt buffer [30 mM Tris-HCl, 10 mM imidazole, 500 mM NaCl and 5% (v/v) glycerol (pH 8.0)], 50 ml of imidazole buffer [30 mM Tris-HCl, 10–30 mM imidazole, 150 mM NaCl, 5 mM β-mercaptoethanol (β-ME) and 5% (v/v) glycerol (pH 8.0)] and 20 ml of 20 mM Tris-HCl (pH 8.0). Finally, bound protein was eluted in elution buffer [20 mM Bis-Tris (Bis-(2-hydroxyethyl) amino-tris(hydroxymethyl) methane), 50 mM NaCl, 10% glycerol and 300 mM imidazole (pH 7.4)] [45]. All purification steps were performed at 4°C. The purity of WT NS and H138C eluted products were monitored on 10% SDS-PAGE. Eluted protein was buffer exchanged and loaded onto an anion exchange HiTrap Q HP column (GE Healthcare) pre-equilibrated with 20 mM Bis-Tris (pH 6.9). Elution of the bound protein was done with different NaCl gradient concentration [20 mM Bis-Tris, 0–1000 mM NaCl, 5% glycerol (pH 6.9)]. Pure protein fractions were pooled, desalted by using the NAP-10 column (GE Healthcare) and concentrated by an amicon centrifugal filter (Merck, Millipore) of 10 kDa cutoff. Monomeric purified protein was analyzed by Coomassie blue staining of 10% SDS/PAGE gels.

### Aggregation assay

WT NS, H138C and W154C-H138C aggregates were prepared by incubating the protein (0.5 mg/ml) in 20 mM Hepes (pH 7.4) at 45°C for 240 min [46]. The incubated sample aliquots were taken at different time points and mixed with an equal volume of loading buffer (250 mM Tris-HCl, pH 6.8, 50% glycerol and 0.5 w/v bromophenol blue). The sample was immediately frozen at −80°C. The presence of the aggregates was confirmed by 10% non-denaturing PAGE and visualized by silver staining. For silver staining gels from SDS-PAGE were fixed for 1 h with 40% methanol, 10% acetic acid and followed by three-time washing at 20 min intervals, gel was incubated with 0.02% sodium thiosulfate for 1 min followed by three quick water rinses. Gel was further incubated for 20 min with cold 0.2% silver nitrate solution and 0.02% formaldehyde followed by two quick water rinses and finally developed in 2% sodium carbonate and 0.05% formaldehyde.

### Transmission electron microscopy (TEM)

For TEM measurement 10 μM WT NS and cysteine variants were incubated for 0, 5, and 240 min at 45°C. About 10 μl of each sample were applied on a carbon-coated grid followed by the negative staining with 1.5% uranyl acetate. TEM images were observed on TECNAI TEM G2 (FEI, electron optics, Netherland) operated at 200 kV.

### Intrinsic fluorescence spectra

The intrinsic fluorescence spectra of WT NS, H138C and W154C-H138C protein was detected on a Jasco FP-6300 spectrofluorometer at a concentration of 2 μM. Protein samples were excited at 280 nm, and the emission spectra were recorded ranging from 300 to 450 nm. The slit widths were 5 nm. The experiments were carried out at room temperature (25 °C) in 20 mM sodium phosphate buffer (pH 7.4), while all required background corrections were made by subtracting a buffer spectrum.

### 4,4-Bis-1-anilino naphthalene 8-sulfonate (Bis-ANS) fluorescence

Bis-ANS is an extrinsic fluorescence probe used to assess the solvent-accessible apolar region on the surface of the protein and give fluorescence just after binding to the exposed hydrophobic region of the protein [47]. To assess the change in surface hydrophobicity, 2 μM WT NS and cysteine variants were incubated at 25°C with Bis-ANS (Sigma, St. Louis MO, U.S.A.) at a 1:5 ratio in dark [13]. An excitation wavelength of 390 nm was used to detect the Bis-ANS fluorescence intensity measurement [48]. Spectra were recorded from 400 to 650 nm on spectra Max M2 Microplate Readers (Molecular Devices, U.S.A.).

### Circular dichroism study

Circular dichroism (CD) experiment was conducted on J-815 spectrometer (Jasco, Japan). For the CD measurement, samples (0.2 mg/ml) in 20 mM sodium phosphate buffer (pH 7.4) were added to a 1 mm path length quartz cuvette. Spectra were collected with a scan speed of 100 nm/min and were averages of three scans. The spectra data of WT NS, H138C and W154C-H138C protein were monitored from 195 to 260 nm.

### Fluorescence labeling of NS cysteine variant

NS cysteine variant H138C was used for site-specific labeling of cysteine by Alexa fluor 488 C_5_ maleimide dye (Molecular probes). Before labeling, protein was incubated in labeling buffer (20 mM HEPES and 100 mM NaCl, pH 7.4) with a 10- to 20-fold excess of TCEP (Thermo Scientific) to maintain the reducing form and prevent cysteine inactivation [49]. About 10 mM dye stock was prepared by dissolving the dye in 100% DMSO solution. For labeling with Alexa Fluor 488 C_5_ maleimide, a total protein concentration of 10–20 µM and a 5- to 10-fold excess of dye was used. Further reaction mixture was incubated in dark at 25°C for 2–3 h and labeling reaction was allowed to proceed overnight at 4°C. The labeling reaction was stopped by using an excess of glutathione (1–2 mM). Free unlabeled fluorophore was removed by using the NAP-10 column (GE Healthcare) pre-equilibrated with 20 mM Hepes and 100 mM NaCl (pH 7.4). Protein dye mixture was loaded on the NAP-10 column and further eluted with the same buffer. The labeling efficiency of the labeled cysteine variant was calculated by observing the absorption spectra of labeled protein using a UV-Vis scanning spectrophotometer (Hitachi, Japan). Different spectral parameter of protein and dye was used to determine the extent of labeling as mentioned below.

**Table:** Parameters of protein and dye used to determine extent of labeling

The molar extinction coefficient of Alexa Fluor 488 C_5_ maleimide	ε_dye_ 77,000 M^−1^ cm^−1^
Absorbance of Alexa Fluor 488 C_5_ maleimide at *λ*_max_ (wavelength of maximum absorbance)	*A*_max__(__dye__)_ 493 ± 3 nM
The molar extinction coefficient of labeled H138C protein	ε_280__(__H__138__C__)_ 37,360 M^−1^ cm^−1^
The absorbance of labeled H138C protein at 280 nM	*A*_280(__H__138__C__)_
*C.F*._280_ of AF 488 C_5_ maleimide*	0.11

a *C.F._280_ *Correction factor* is the ratio of absorbance of free dye at 280 nm and at its *λ*_max_ [49,50].

The degree of labeling for labeled NS single cysteine variants was calculated by using the following formula [50,51].

(1) $$D.O.L.=\frac{\varepsilon_{280(H138C)}.A_{max(dye)}}{(A_{280(H138C)}-C.F._{280}A_{max(dye)})\cdot \varepsilon_{dye}}$$

The concentration of labeled NS single cysteine variants *C*(*M*) were calculated by

(2) $$C(M)=\frac{\left(A_{280(H138C)}-C.F._{280}\;A_{max(dye)}\right)}{\varepsilon_{280(H138C)}}$$

For conformational analysis, 150 nM labeled H138C protein was incubated at 25°C for complex formation for 10 min with various concentration of tPA (> 85% sc-tPA, Molecular Innovations, U.S.A.) in 50 mM HEPES (pH 7.4), 150 mM NaCl and 0.1% Tween-20. Fluorescence emission spectra of Alexa Fluor 488 C_5_ maleimide labeled protein were monitored on Spectra Max M2 Microplate Readers (Molecular Devices, U.S.A.). A 480 nm excitation wavelength for dye was used and emission spectra of dye-bound protein were recorded from 500 to 650 nm. All fluorescence spectra were repeated three times and corrected by subtracting the buffer spectra.

### NS-tPA activity assay

Inhibition of tPA by WT NS, H138C, W154C-H138C (0–800 nM) and labeled H138C protein (0–400 nM) were monitored at 405 nm absorbance in the presence of 800 µM chromogenic substrate CH_3_-SO_2_-D-HHT-GLY-ARG-P-NITROANILIDE.AcOH (T-2943, Sigma-Aldrich, St. Louis MO, U.S.A.) [45]. NS and tPA substrate were added to the microtiter plate followed by the immediate addition of a constant amount of 19 nM tPA. The progressive curve for WT NS and cysteine variants (including labeled H138C) were observed for 120 min by the release of pNA upon hydrolysis of chromogenic substrates by the tPA [52]. Control protease activity was measured using the same method except for the sample containing no NS. All kinetic experiments were performed at 25°C in kinetic buffer [50 mM Hepes, 150 mM NaCl and 0.1% Tween-20 (pH 7.4)] on spectra Max M2 Microplate Readers (Molecular Devices, U.S.A.).

### NS-tPA enzyme complex formation

For the complex formation, tPA (250 nM) was mixed with purified WT NS, H138C and W154C-H138C (750 nM). After incubation at room temperature for varying periods, an aliquot (25 μl) of the reaction was withdrawn and stored at −80°C. To measure Alexa fluor C_5_ Maleimide labeled H138C-tPA complex formation, aliquots of the reaction containing H138C (150 nM) and tPA (150–200 nM) were incubated for 10 min at 25°C and stored at −80°C. The reactions were stopped by adding reduced SDS-loading buffer (125 mM Tris-HCl, 4% SDS, 10% [w/v] β-ME, 20% glycerol, 0.2 w/v bromophenol blue) at 95°C and immediately heated for 10 min. SDS-gel electrophoresis (10%) was performed for complex formation analysis and the gel was visualized by Nimble juice speedy protein gel stain (Gene direx).

## Result

### Molecular simulation analysis of strand 1A variant

**Figure 2:**
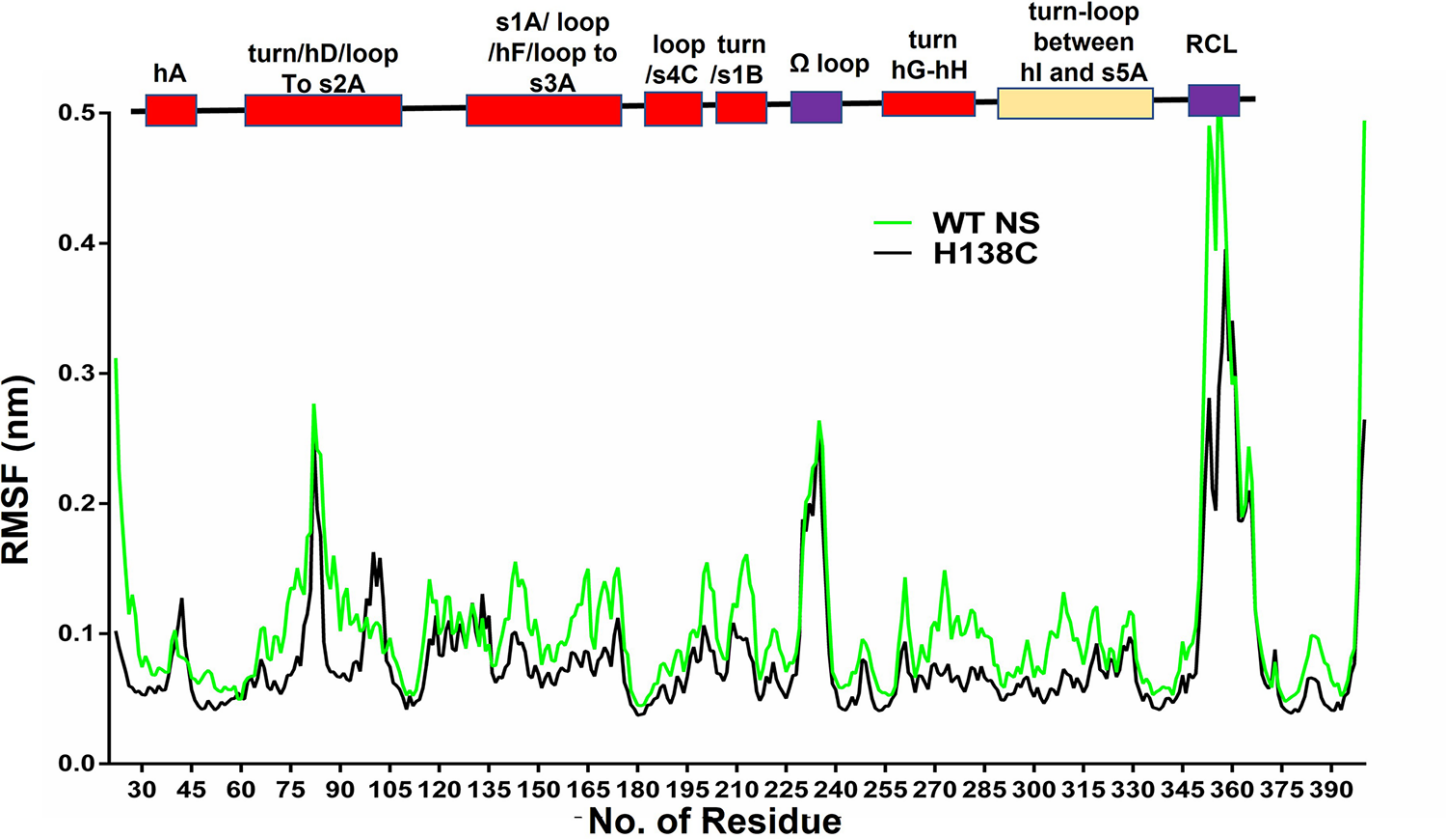
Root mean square fluctuation (RMSF) plot RMSF comparison of the Cα atoms for each residue of WT NS (green) with H138C (black) protein over the 50 ns of the trajectory.

MD simulation was done to explore the changes in the conformational dynamics of NS strand 1A variant using GROMACS simulation software. A RMSF (Root-mean-square fluctuations) plot of H138C (Figure 2) shows several regions including the RCL that shows reduced fluctuations, and also a decreased fluctuation in the s1A-loop to helix F and helix F-loop to the s3A region (135–178). The difference between the Cα backbones of H138C measured by (root mean square deviation) RMSD (Supplementary Figure S1A) indicated a smaller structural deviation. A marginally higher solvent accessible area SASA value (Supplementary Figure S1B) of the cysteine variants was observed as compared with WT NS. The radius of gyration (*R*_g_) is the root mean square distance of a group of atoms from their common center of mass indicate the level of protein compactness and a lowest *R*_g_ value indicated the tight packing [53] (Supplementary Figure S1C).

### Expression and purification of strand 1A NS variant

Mutation in the NS strand 1A was incorporated using Stratagene QuikChange II site-directed mutagenesis Kit with mutagenic primer (Supplementary Figure S2) and one-step PCR [54]. Amplification was checked by electrophoresis on a 0.8% agarose gel and by Sanger sequencing (Supplementary Figure S2A and S2B). The purified plasmid of strand 1A was transformed in BL21 (DE3) cells for protein expression. NS gene carrying the N-terminal hexa-histidine tag under the control of the T7 promoter in the pET 28b vector was purified by Ni-NTA affinity (Supplementary Figure S3A and S3B) and anion-exchange chromatography (AEC) (Supplementary Figure S3C and S3D). All monomeric fractions were collected and concentrated to the desired level and stored at −80°C.

### Mutations in the neuroserpin strand 1A residue increases its aggregation propensity

**Figure 3:**
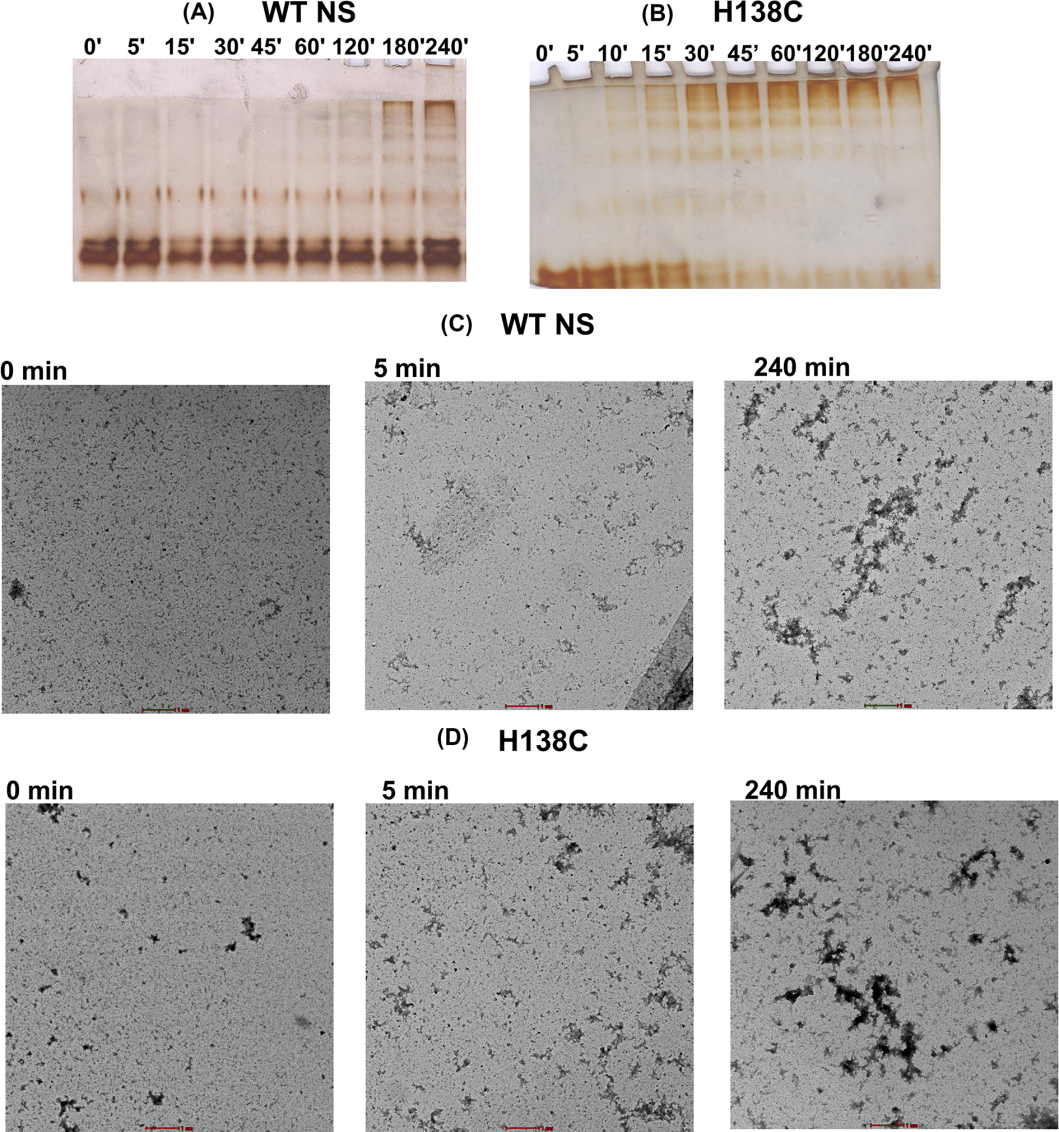
Aggregation analysis of strand 1A variants (**A**) Aggregation profile of WT NS and (**B**) H138C. The representative transmission electron microscope micrograph of (**C**) WT NS, and (**D**) H138C is shown at 0, 5 and 240 min. The scale bar represents 1 μm.

Neuroserpin readily form aggregates at a slightly higher than physiological temperature, its shutter region is highly prone to aggregation as all the FENIB mutation has been identified in or near this region [9,16,17], NS aggregation propensity was examined by using non-denaturing PAGE and TEM. WT NS forms oligomers after 30 min of incubation, but high molecular weight aggregates form after 60 min of incubation (Figure 3A). In contrast, the H138C variant forms high molecular weight aggregates after 10 min. Interestingly the high molecular weight aggregates band intensity of the H138C variant increases at 30 min followed by the complete conversion of lower molecular weight aggregates to high molecular weight aggregates after 45 min incubation (Figure 3B). TEM analysis was performed to confirm the morphology of the aggregates of WT NS and the cysteine variant. About 10 µM of WT NS and H138C variant were heated at 45°C for 0, 5 and 240 min (Figure 3C,D). At the end of incubation (240 min), more intense aggregates were observed in the H138C variant (Figure 3D) as compared with the WT NS (Figure 3C), agreeing with results obtain using non denaturating PAGE.

### Conformational analysis of strand 1A variant

**Figure 4:**
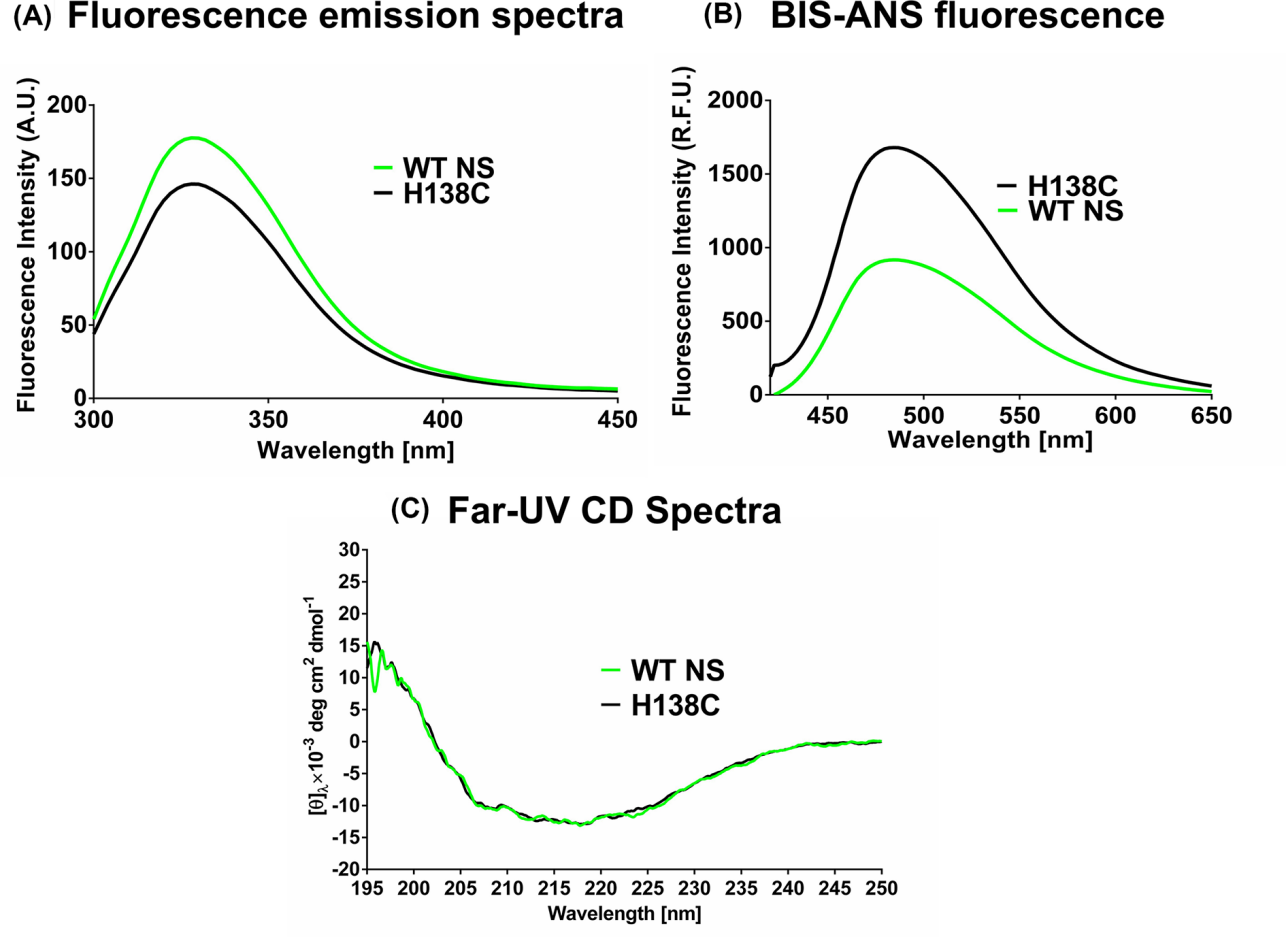
Conformational analysis of strand 1A variant (**A**) Changes in the intrinsic fluorescence intensity of 2 μM WT NS (green) and H138C (black) λ_exc_ (280 nm). (**B**) Bis-ANS fluorescence spectra indicate the relative amount of surface hydrophobicity for WT NS (green) and H138C (black), λ_exc_ (390 nm). (**C**) The figure represents secondary structure analysis with 0.2 mg/ml WT NS (green) and H138C (black) using far-ultraviolet circular dichroism (CD) spectra in the range of 195–250 nm. Each graph is an average of at least three independent plots.

Intrinsic fluorescence of s1A cysteine variant (H138C) and WT NS (2 μM) was studied to detect the change in conformation at the level of tertiary structure. Fluorescence spectra were recorded in 20 mM sodium phosphate buffer (pH 7.4). A decrease in fluorescence intensity was observed in the s1A variant suggesting internalization of Tyr and Trp residues representing a more compact nature of the variant (Figure 4A) as compared with WT NS. The exposure of the hydrophobic residues on the surface of the H138C variant (2 μM) was monitored by observing the Bis-ANS (10 μM) binding. More hydrophobic exposure was observed due to increase Bis-ANS fluorescence in the H138C variant (Figure 4B). Secondary structural changes were compared using circular dichroism (CD) spectra in 20 mM sodium phosphate buffer (pH 7.4) at 0.2 mg/ml concentration. Far-UV CD spectra of the H138C variant showed comparable results indicating that the secondary structure profile is largely maintained in the variant (Figure 4C).

### Strand 1A cysteine variant is an active tPA-inhibitor

**Figure 5:**
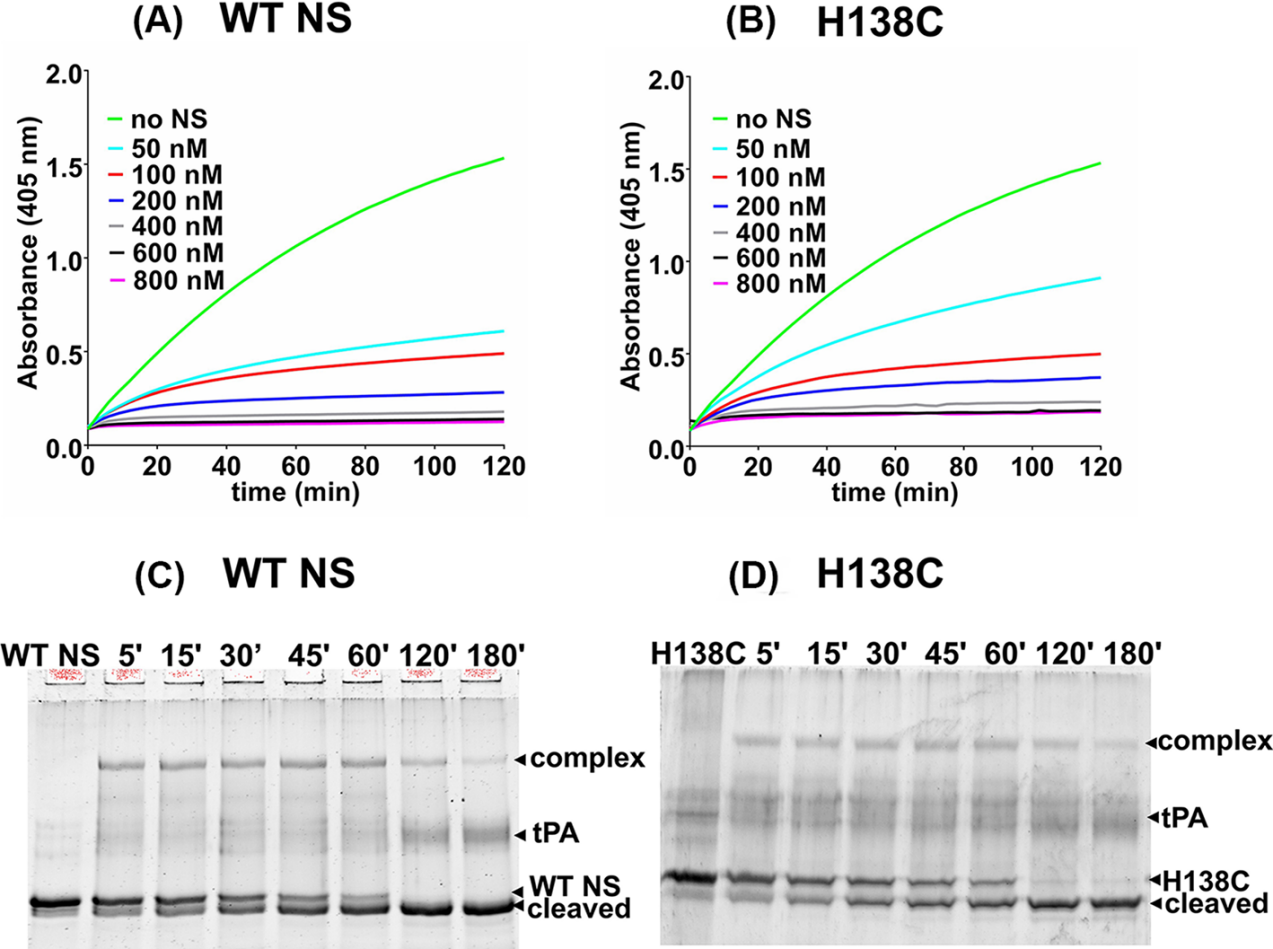
tPA inhibition activity and complex formation assay (**A**) Progress curves of the hydrolysis of the substrate (800 μM) by tPA (19 nM) in the presence of NS concentration (0–800 nM) of WT NS and (**B**) H138C protein; 0 nM (green), 50 nm (cyan), 100 nM (red), 200 nM (blue), 400 nM (gray), 600 nM (black), and 800 nM (pink) represent concentration of NS. SDS-PAGE (10%) showing time-dependent analysis of complex formation between 750 nM (**C**) WT NS and (**D**) H138C with 250 nM tPA. Arrows indicate the different species: NS-tPA complex, tPA, native and cleaved NS. Each graph is an average of more than three independent plots.

NS forms complexes with tPA *in vitro*, however, NS is a transient inhibitor of tPA in the brain as this interaction is short-lived that eventually releases the active tPA [52,55]. The inhibitory activity of WT NS and H138C with tPA was analyzed utilizing a tPA chromogenic substrate. In the presence of the WT NS and H138C variant, a progressive increase in the rate of substrate hydrolysis by tPA was observed after the hydrolysis of IPR-pNA (Figure 5A). Inhibitory activity of the H138C variant was found to be comparable to WT NS (Figure 5B). The ability of WT NS and H138C to form a complex with tPA was also analyzed by incubating NS and tPA in a 3:1 molar ratio at different time intervals. NS-tPA complex remained stable in WT NS for up to 60 min (Figure 5C). A stable complex formation was also observed between H138C and tPA (Figure 5D). WT NS and H138C showed the increased intensity of the cleaved band with decreased intensity of the NS native band. tPA inhibition profile and ability to form tPA complex were not altered in the strand 1A variant indicating a marginal effect on the inhibition mechanism.

### Site-specific conformation analysis of AF 488 C_5_ maleimide labeled H138C variant

**Figure 6:**
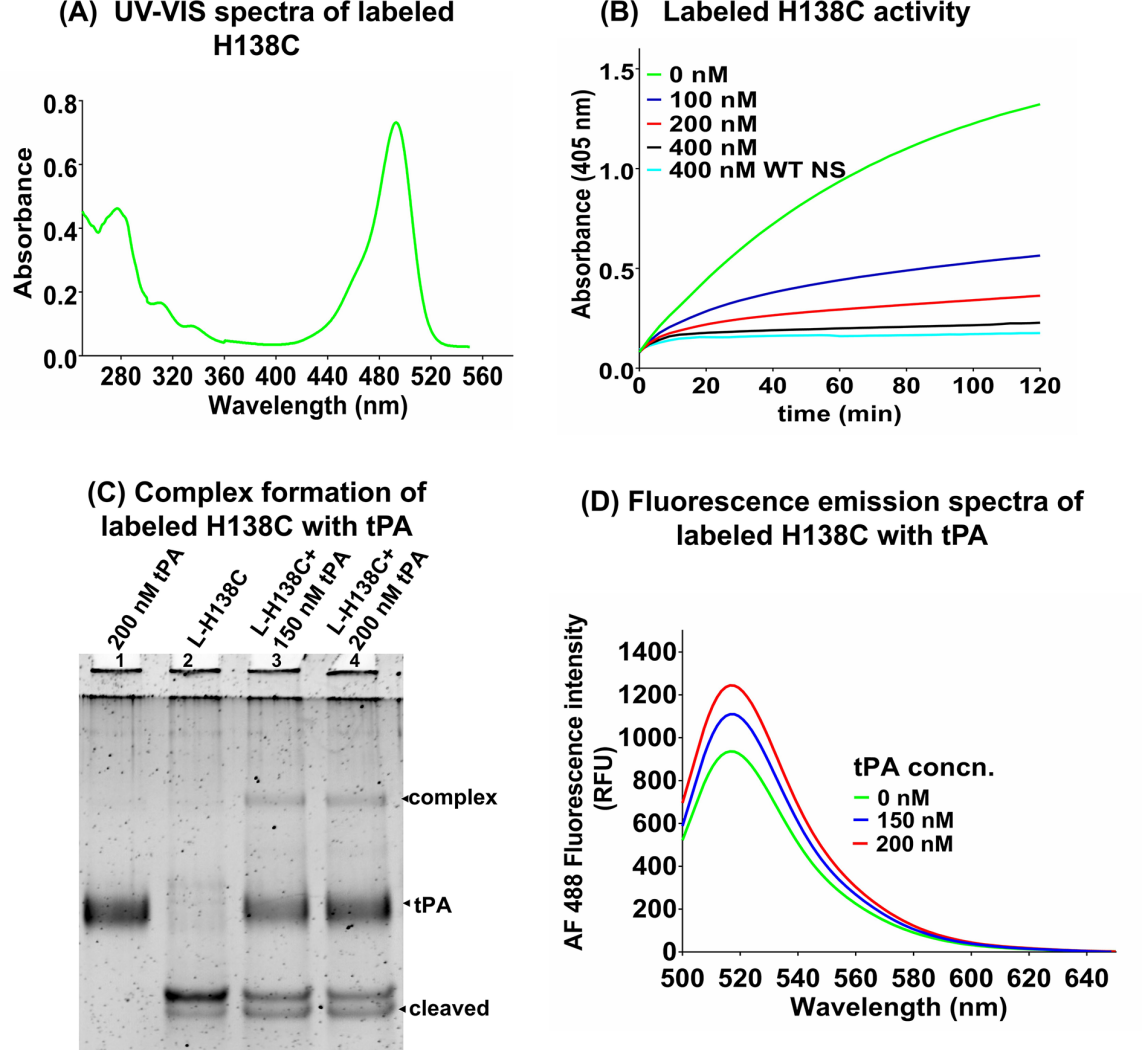
Characterization of the labeled H138C (**A**) UV-Vis spectra analysis of AF 488 C_5_ maleimide labeled H138C represented the protein and dye peak at 280 and 493 nm, respectively. (**B**) tPA inhibition activity assay of AF 488 C_5_ labeled H138C showing the progressive curve of labeled H138C at a concentration of 0–400 nM. About 400 nM NS (cyan) was used as a control for labeled H138C activity. (**C**) SDS-PAGE (10%) gel showing the complex formation of 150 nM labeled H138C with 150 nM tPA (lane 3) and 200 nM tPA (lane 4). Lanes 1 and 2 correspond to tPA and labeled H138C as a control. (**D**). The relative changes in the fluorescence emission spectra of AF 488 C_5_ maleimide labeled H138C (150 nM) in the absence (red line) and in the presence of 150 nM (blue), 200 nM (red) tPA. Each experiment was repeated several times and the graph represents the average of at least three observations.

During the inhibition mechanism, the insertion of RCL into the NS β-sheet A requires conformational changes in the β-sheet A strand and helix F [11,12]. To assess site-specific modification on the inhibition parameters and conformation, H138C was labeled with AF 488 C_5_ maleimide as described in the ‘Material and Methods’ section. Absorption spectra showed that H138C was site-specifically labeled and showed characteristic absorbance of the AF 488 C_5_ dye (Figure 6A). The degree of labeling for the labeled H138C was calculated to be ∼0.92 by using eqn 1. To understand the effect of H138C labeling on tPA inhibition, we examined its tPA-inhibitory activity and complex formation ability (Figure 6B,C). Inhibitory activity of the H138C variant was found to be comparable to WT NS (Figure 6B), and a stable complex formation was also observed between the labeled H138C and tPA with the appearance of the cleaved band (Figure 6C). Further dye-labeled H138C was used to determine conformational changes in strand 1A of β-sheet A due to the tPA binding (Figure 6D). An increase in the fluorescence intensity of the dye with the increase in the concentration of the tPA was observed indicating a solvent exposure of the dye bound H138C in strand 1A of β-sheet A. The result indicated that labeling of H138C does not affect the complex formation with tPA and results in the exposure of the s1A.

### Interplay between strands 1A and helix F mutant impacts neuroserpin stability

**Figure 7:**
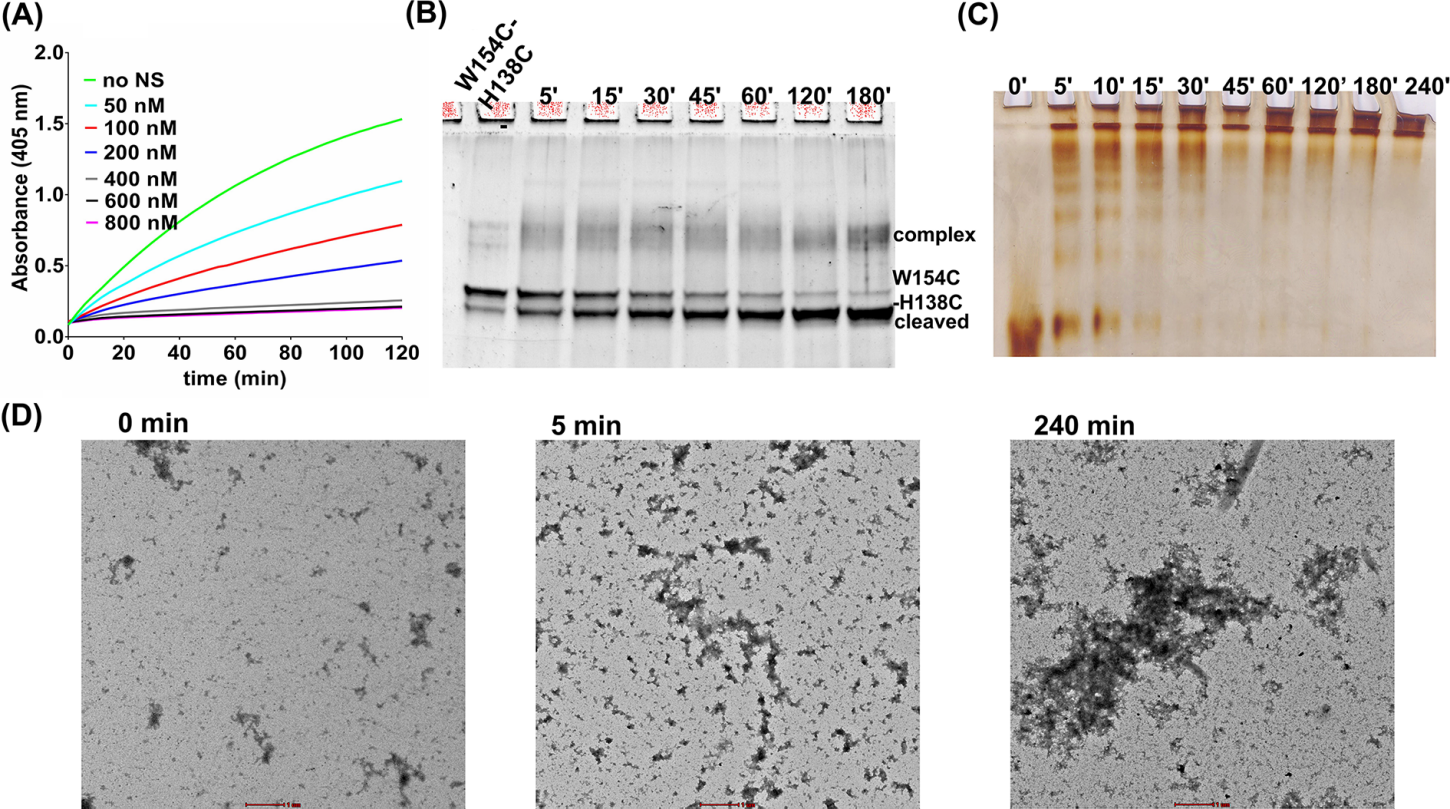
Biochemical characterization of W154C-H138C (**A**) Progress curves of the hydrolysis of substrate (800 μM) by tPA (19 nM) in the presence of W154C-H138C protein. 0 nM (green), 50 nm (cyan), 100 nM (red), 200 nM (blue), 400 nM (grey), 600 nM (black), 800 nM (pink) represent concentration of NS. (**B**) 10% SDS PAGE showing time-dependent analysis of complex formation between 750 nM W154C-H138C with 250 nM tPA. (**C**) 10% non-denaturing PAGE showing aggregation profile of W154C-H138C (**D**) Transmission electron microscope images of W154C- H138C at 0, 5 and 240 min.

Strand 1A C-terminal connects to helix F through a loop and can influence its conformation during tPA-bound RCL insertion (Figure 1). We used W154C plasmid DNA to incorporate a W154C-H138C double mutation by using the mutagenic primer. Amplification was checked by electrophoresis on a 0.8% agarose gel and sequencing was performed to confirm the variant (Supplementary Figure S4A and S4B). W154C-H138C protein was purified by Ni-NTA affinity and anion-exchange chromatography (AEC) and monomeric fraction were confirmed by SDS-PAGE (Supplementary Figure S5A and S5B). The double variant showed a decreased fluoroscence emission intensity, and a very high surface hydrophobicity, although its secondary structure profile matched with the native NS (Supplementary Figure S6). W154C-H138C protein showed a slight decrease in the tPA inhibition activity and also the complex formation with tPA was also compromised (Figure 7A,B). This protein was further analyzed for the aggregation propensity and instantly form high and low molecular weight aggregates (Figure 7C). However, all the native proteins disappear after 15 min due to conversion to higher molecular weight aggregates. Consequently, TEM analysis of the double variant showed higher intensity of lower and higher molecular weight oligomers at 5 min of incubation (Figure 7D), which converts to highly intense amorphous aggregates at 240 min incubation. These results implicate the role of helix F and strand 1A in the increased propagation of NS aggregates, with marginal affects on the tPA inhibition rates.

## Discussion

Serpin inhibitory mechanism is an excellent example for understanding a well-coordinated conformational transition that acts as a trap for protein inactivation. Unfortunately, this elegant mechanism also makes serpins prone to conformational defects leading to pathological phenotype and giving rise to diseases like thrombosis, emphysema, cirrhosis, angioedema, epilepsy, and dementia. The domains with large conformational variations like helix F, strand 1A, 2A, 3A, and 5A, have been identified through crystal structures, simulation and HXMS studies. The nature of interaction and conformational changes during the inhibition mechanism and the polymer intermediates and their role in forming the aggregates is not well understood [12,15]. Helix F has been predicted to unfold at both N and C terminal end during RCL transition, but another hypothesis predicted helix F to be displaced and rearranged after RCL transition [12,31,32]. Variants in the helix F, strand 6B, helix B and strand 2A in the shutter regions show appreciable increase in the polymerization, however in these variants inhibitory activity against tPA is largely maintained [13,44,45]. Strand 1A is away from the RCL insertion site however it is connected to the helix F through a loop and its N-terminal end connects to s2A through helix E (Figure 1), strand 1A variants can influence the inhibition mechanism, unlike other shutter region variants that retain inhibition activity.

A 50 ns molecular simulation study of comparison between H138C and WT NS showed several differences in the conformation of the variant (Figure 2). A higher value of RMSD was observed in H138C which indicated that this variant is destabilized as compared with WT NS (Supplementary Figure S1A), but the radius of gyration indicated higher compactness in H138C (Supplementary Figure S1C). H138C RMSF value showed decreased mobility in the loop connecting s1A to helix F and loop connecting helix F to s3A region (Figure 2). H138C variant was mutated, expressed and purified to homogeneity using Ni-NTA and anion exchange chromatography (Supplementary Figure S3A and S3B). The secondary structure of the variant was maintained, although the tertiary structure and surface hydrophobicity showed marked variations as compared with the WT NS control (Figure 4). Phenyl group shielding and decrease in the ensuing fluorescence indicate a more compact structure in the H138 variant agreeing with *in silico* results. Site-specific labeling of the strand 1A variants with AF 488 C_5_ maleimide (Figure 6A) showed an increase in the fluorescence intensity in H138C on increase in the concentration of tPA (Figure 6D), indicating that strand 1A is exposed during the inhibition mechanism. The variant showed inhibition of tPA and was able to form complexes both in the labeled or unlabeled form indicating tPA inhibition mechanism is retained (Figures 5 and 6B,C).

The aggregation profile of H138C shows high molecular weight aggregate formation on native PAGE after 60 min of incubation (Figure 3A). Interestingly, the H138C variant starts to form oligomer after 10 min of incubation, which decreases the intensity of lower molecular weight aggregates with a progressive increase band intensity of high molecular weight aggregate (Figure 3B,D) as compared with WT NS (Figure 3C).

A known variant of helix F was introduced and the W154C-H138C helix F-strand 1A double variant formed oligomeric and high molecular weight aggregate instantly (Figure 7C,D). However its tPA inhibitory activity was maintained (Figure 7B). Labeled H138C and W154C-H138C double variants are more severe form of variants but the inhibitory activity is only marginally compromised (Supplemenatry Figure S7). A number of recent studies with shutter region variants indicate that although the variants readily form polymers, they retain appreciable tPA inhibition activity and the ability to form complexes [45,46]. It seems that small molecules that crosses blood–brain barrier and can bind to the shutter region of the NS and can hinder the polymer formation and will be able to retain appreciable tPA inhibition acitivity; therefore, NS-based pathologies can be corrected.

## Conclusion

Polymerization of the NS is related to variable pathological phenotype based on the variation in the type of NS variants [16,17]. A number of NS variants that polymerize easily on change in the condition also retain appreciable inhibitory activity. Therefore, blocking the oligomerization of such variants will significantly retain its inhibitory activity and is expected to improve the disease phenotype. Cell based *in vivo* studies of NS polymerization using a Zebrafish type model and effective polymerization inhibition using an embelin type of molecule will significantly help in the understanding of ameilorating NS pathological phenotype [56,57].

## Supplementary Material

Supplementary Figures S1-S7

## Data Availability

All the supporting data are included within the main article and the supplementary files.

## Abbreviations

AEC: anion-exchange chromatography
Bis-ANS: 4,4-bis-1-anilino naphthalene 8-sulfonate
CD: circular dichroism
MD: molecular dynamics
RCL: reactive center loop
RMSF: root mean square fluctuation
