# Supplementary material for: Strand 1A variant in neuroserpin shows increased aggregation and no loss of inhibition: implication in ameliorating polymerization to retain activity
Source: Biosci Rep. 2022 Dec 16;42(12):BSR20221825. doi: 10.1042/BSR20221825 (PMC9760604; doi:10.1042/BSR20221825)
Supplement: Supplementary Figures S1-S7 [file BSR-2022-1825_supp.pdf]

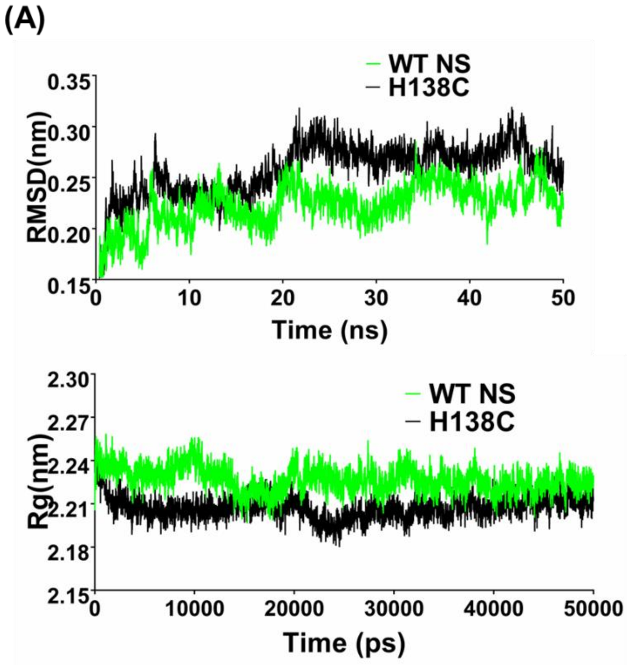


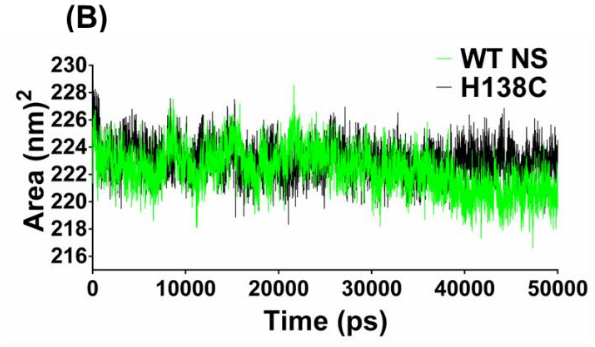


| MD studies | Average RMSD (nm) | SASA ($nm^2$) |
| --- | --- | --- |
| **WT NS** | 0.222±0.01 | 222.31±0.02 |
| **H138C** | 0.257±0.02 | 223.05±0.01 |

**Supplementary Figure S1. Structural parameters analyzed from molecular dynamics simulations. (A)** The Root mean square deviation (RMSD) of carbon α backbone atoms, (B) Solvent accessible surface area (SASA) and (C) The radius of gyration (Rg) vs time over the course of the entire 50 ns simulation of WT NS and cysteine variants. Green; WT NS and black; H138C. Molecular simulation parameters for WT NS and H138C showing the average value of Cα root mean square displacement (RMSD) and solvent accessible surface area (SASA) are given as inset.

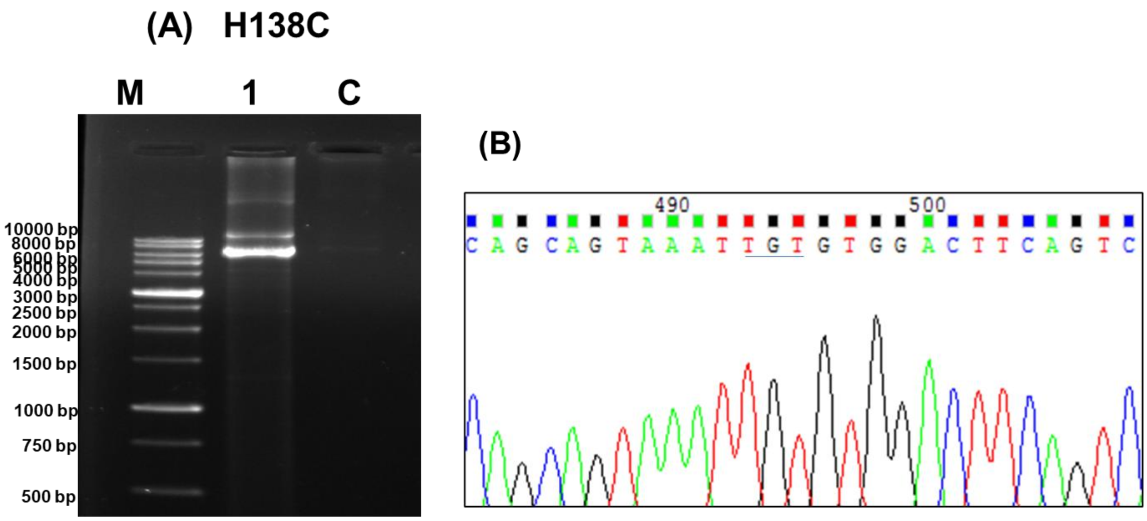


| Neuroserpin mutant oligo | Template/Primer | | Primer sequence (5`-3`) | Tm (°C) |
|---|---|---|---|---|
| H138C (s1A) | W154C-H138C/C154W | F | CTACATCAATAAGTG**G**GTGGAGAATAACAC | 57.0 |
| | | R | GTGTTATTCTCCAC**C**CACTTATTGATGTAG | |

**Supplementary Figure S2. Site-Directed Mutagenesis of H138 residue. (A)** 0.8% agarose gel detection of site-directed mutagenesis reaction H138C, Lane1- PCR amplified product at 55°C annealing temperature. Lane C-control, lane M represents the DNA ladder standard (Gene direx). Sequencing electropherograms confirm the **(B)** H138C mutation. The mutation is indicated by the black horizontal line. Primer sequences used for the site-directed mutagenesis are given as inset.

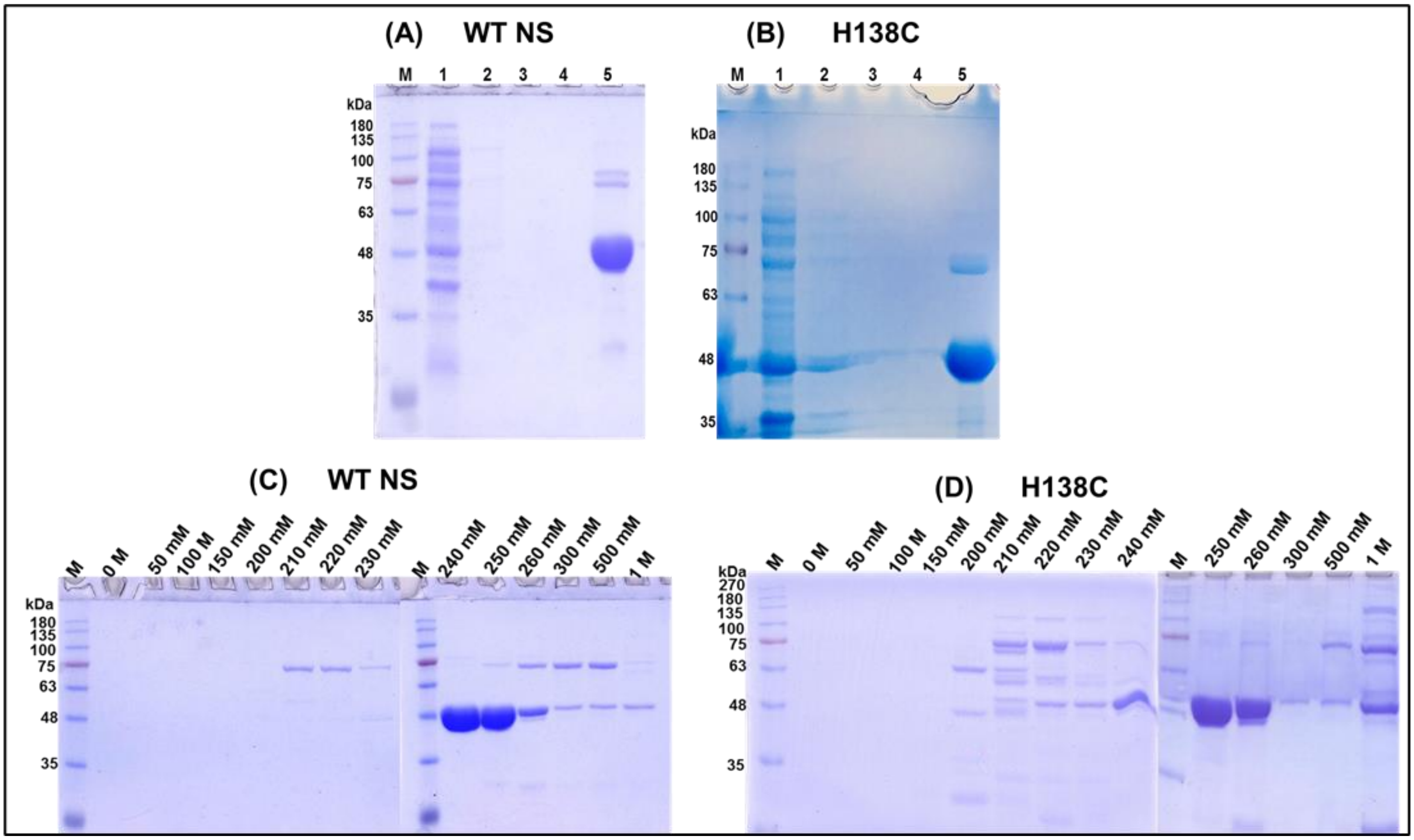


**Supplementary Figure S3. Purification profile of WT NS and H138C protein. (A)** 10 % SDS-page analysis of affinity-purified WT NS and **(B)** H138C. Lane 1-4 corresponds to unbound sonicated cell lysate, washing with salt buffer, imidazole buffer and tris HCl. Purified native or mutant NS eluted using elution buffer with 300 mM imidazole (lane 5). 10 % SDS-PAGE analysis with Coomassie staining of the anion exchange chromatography fraction of **(C)** WT NS and **(D)** H138C.

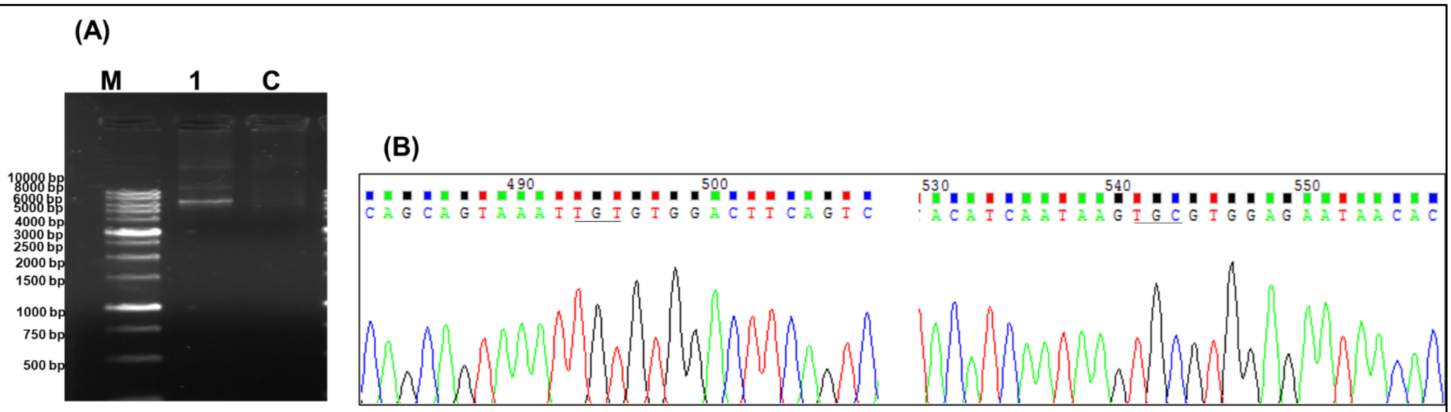


| Neuroserpin mutant oligo | Template/Primer | | Primer sequence (5`-3`) | Tm (°C) |
|---|---|---|---|---|
| W154C-H138C (Helix F-s1A) | W154C/H138C | F | TTTAATGCAGCAGTAAAT**TG**TGTGGACTTCAGTC | 60.5 |
| | | R | GACTGAAGTCCACA**CA**ATTTACTGCTGCATTAAA | |

**Supplementary Figure S4. Site-directed mutagenesis of W154-H138 residue. (A)** 0.8% agarose gel detection of site-directed mutagenesis reaction W154C-H138C, Lane1- PCR amplified product at 55°C annealing temperature. Lane C-control, lane M represents the DNA ladder standard (Gene direx). **(B)** Sequencing electropherograms confirm the W154C- H138C mutation. The mutation is indicated by the black horizontal line. Primer sequence used for the site-directed mutagenesis is given as inset.

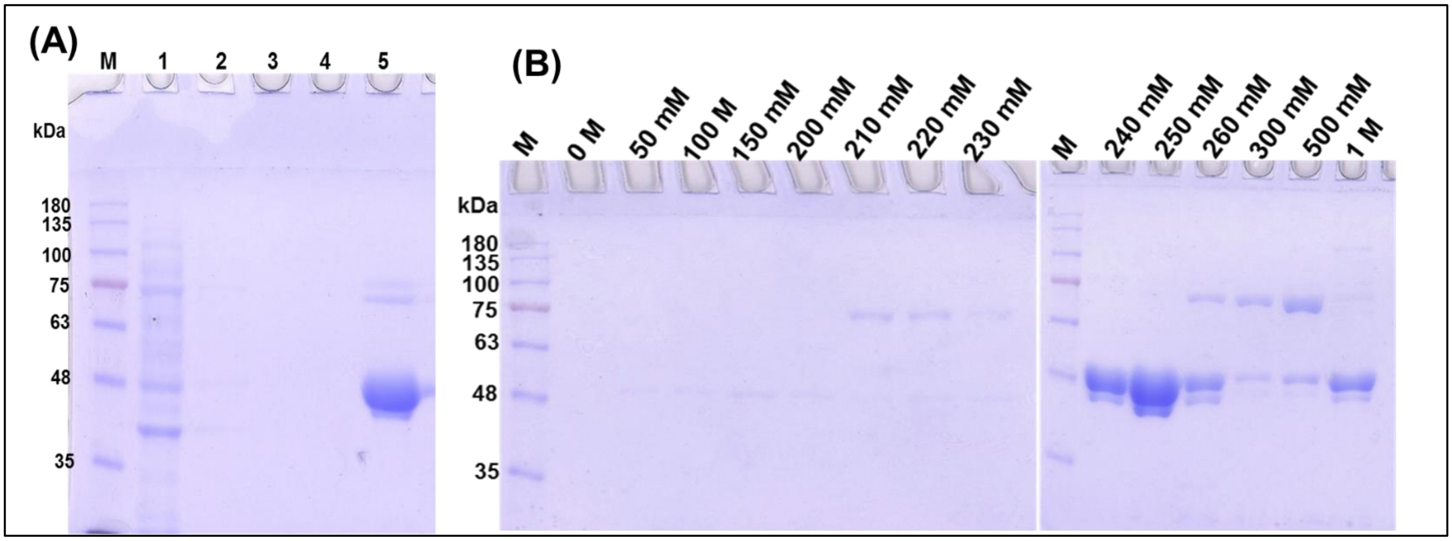


**Supplementary Figure S5. Purification profile of W154C- H138C protein. (A)** 10 % SDS-page analysis of affinity-purified and **(B)** anion exchange chromatography fraction of W154C-H138C.

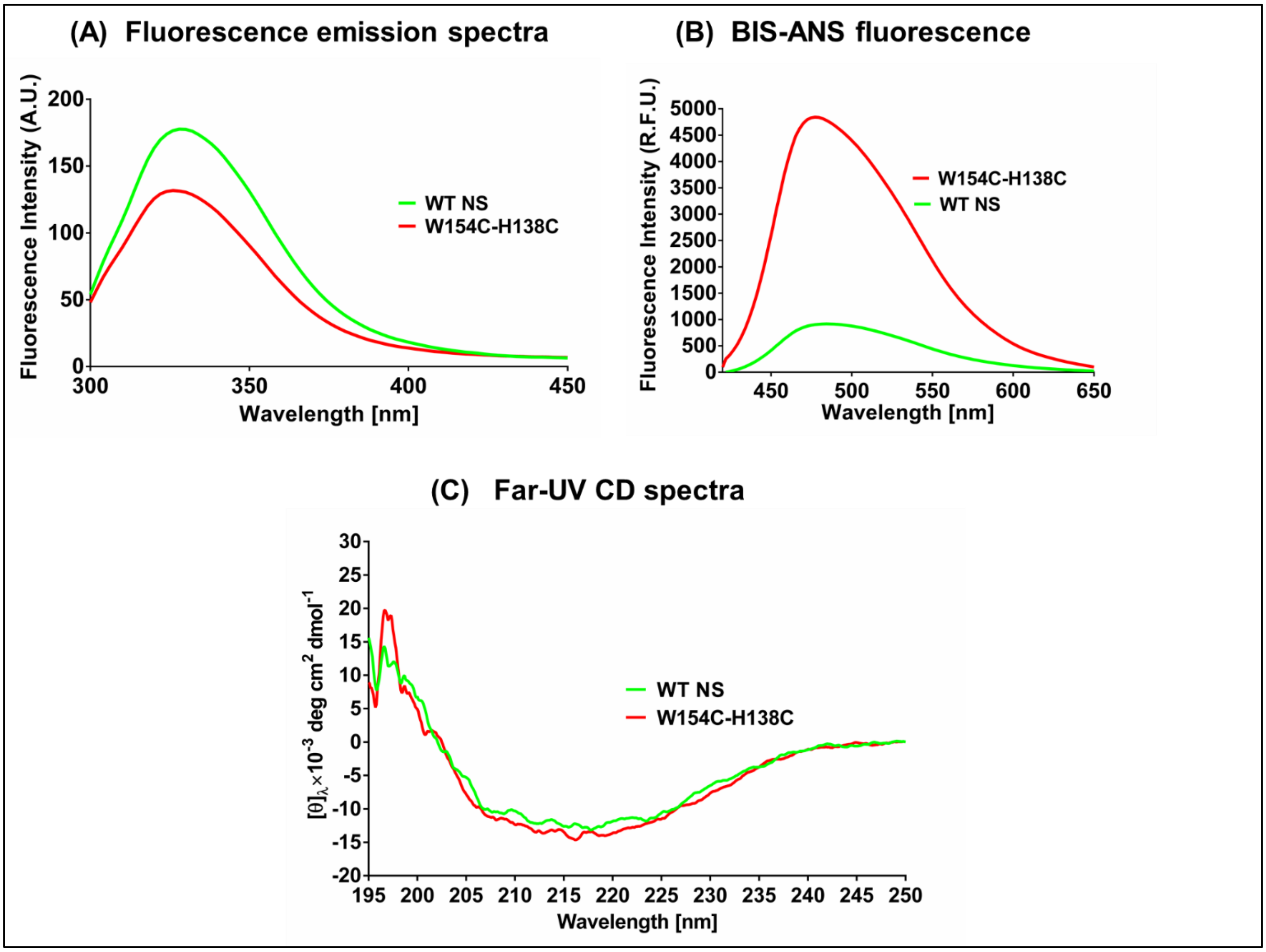


**Supplementary Figure S6. Structural studies of the W154C-H138C double NS variant. (A)** Intrinsic fluorescence. Change in the fluorescence intensity of 2 μM WT NS (green), and W154C-H138C (red). (B) Bis-ANS fluorescence. Bis-ANS fluorescence spectra indicate the relative amount of surface hydrophobicity for WT NS (green) and W154C-H138C (red). (C) CD spectroscopy. Secondary structures analysis of the 0.2 mg/mL WT NS (green) and W154C-H138C (red).

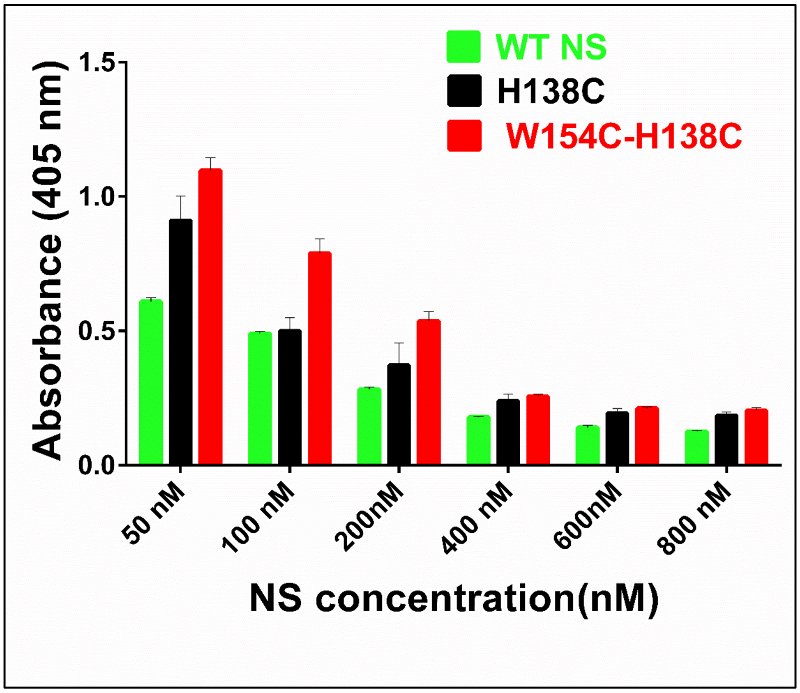


**Supplementary Figure S7. A comparison of the activity of the NS variants and the wild type NS.** Hydrolysis of the substrate (800 µM) by tPA (19 nM) in the presence of WT NS and its variants.
